# Intensity of adoption and welfare impacts of drought-tolerant rice varieties cultivation in Bangladesh

**DOI:** 10.1016/j.heliyon.2022.e09490

**Published:** 2022-05-18

**Authors:** Md. Sadique Rahman, Md. Hayder Khan Sujan, Debasish Chandra Acharjee, Rezoyana Kabir Rasha, Mofasser Rahman

**Affiliations:** a Department of Management and Finance, Sher-e-Bangla Agricultural University, Sher-e-Bangla Nagar, Dhaka, Bangladesh; b Department of Development and Poverty Studies, Sher-e-Bangla Agricultural University, Sher-e-Bangla Nagar, Dhaka, Bangladesh; c Department of Agribusiness, Bangabandhu Sheikh Mujibur Rahman Agricultural University, Gazipur, Bangladesh; d Department of Agricultural Economics, Sher-e-Bangla Agricultural University, Sher-e-Bangla Nagar, Dhaka, Bangladesh; e Department of Agribusiness and Marketing, Sher-e-Bangla Agricultural University, Sher-e-Bangla Nagar, Dhaka, Bangladesh

**Keywords:** Drought, Food grain availability, Productivity, Rice farming, Tobit model, Treatment effect model

## Abstract

Rice production in Bangladesh is vulnerable to climate-related risk such as drought, which contributes to food insecurity. Adoption of drought-tolerant rice varieties can play an important role in increasing productivity, food grain supply, and income. However, to the best of our knowledge, no studies have measured the welfare impacts of drought-tolerant rice varieties in the South Asian and Bangladeshi context. Therefore, this study identifies the factors that influence the intensity of adoption and welfare impacts of drought-tolerant rice varieties in Bangladesh. To accomplish these objectives, 300 rice growers from three drought-prone districts of Bangladesh were surveyed. To analyze the impacts, the entire sample was divided into three groups depending on their share of land under drought-tolerant rice variety cultivation: full adopters, partial adopters, and non-adopters. The descriptive statistics, two-limit Tobit model and multivalued treatment effect models were used to analyze the data. According to the findings, training as well as technology-related factors play a major role in boosting the intensity of adoption. Full adopters of drought-tolerant varieties receive 1222–1473 kg higher yield per hectare compared to non-adopters. Based on several treatment effect models, the impact on income ranges from 3.46% to 4.22%. When compared to non-adopters, full adopters can consume 1.02–1.29 months more rice from their own production in a year. Shows about climate change and other relevant topics should be broadcast on the television on a regular basis to raise awareness. Modifying the extension method with modern communication technologies will aid in widespread adoption of new technologies. Drought-tolerant rice varieties can help to mitigate the harmful effects of drought and alleviate poverty in drought-prone areas.

## 1 Introduction

Rice is a staple food for more than half of the world's population and the primary source of income for 20% of the world's population (Mottaleb et al., 2014; Dar et al., 2020). During the triennium ending 2018 over 1963, worldwide rice consumption climbed from 40.70 kg per capita to 52.69 kg per capita (Samal et al., 2021). As a result, by 2050, worldwide rice demand will reach 584 million tons (Samal et al., 2021). In recent years, rice demand has also increased in Asian countries (Mattaleb et al., 2014). Climate change, however, negatively affects rice production throughout the world, jeopardizing food supplies. Drought, a climate-related hazard, produces the most damaging effects on rice production. Drought significantly reduces rice grain yield as well as vegetative growth (Dar et al., 2020). It has impacted approximately half of the world's rice cultivation area, posing a serious threat to food security (Bouman et al., 2005; Dar et al., 2020).

Drought is a significant impediment to sustainable crop production and food security in Bangladesh. Drought mostly impacts Bangladesh's northwestern region, where 1.2 million hectares of land are used to farm rice during the dry season (Islam et al., 2017). Bangladesh faced severe drought in this area in 1999, 2000, 2006, 2009, and 2012. Bangladesh suffered the longest drought in 50 years in 1999, going more than four months without rain. Crop production decreased by 25%–30% as a result of the prolonged drought, posing a serious threat to food grain supply. Bangladesh's Ministry of Agriculture reported that moderate to extreme drought had affected approximately 57% of the country's total net cultivated land. Nonetheless, owing to the increasing severity of drought and crop production losses, adaptation to drought problems through the use of climate-smart agricultural practices has been emphasized in recent years (Islam et al., 2017).

The Bangladeshi government has implemented drought management initiatives to mitigate the impact of droughts. Farmers in Bangladesh's drought-prone areas can now look forward to a more plentiful rice harvest with the release of many drought-tolerant rice varieties (BRRI dhan 56, BRRI dhan 66, BRRI dhan 71) by the Bangladesh Rice Research Institute (BRRI). Drought-tolerant rice varieties are those that can produce a reasonable yield even when soil moisture is less than 20% and the perch water table depth is more than 70–80 cm from the surface level (Kader et al., 2019). All of these varieties can reach maturity in 105–115 days. Drought-tolerant varieties can produce at least 3.5–5 t/ha without watering throughout the reproductive period (Kader et al., 2019). Traditional rice varieties in Bangladesh wither and die within 10–12 days if water is not available, while drought-tolerant cultivars may survive without rain up to 27 days (Islam, 2011). Drought-tolerant varieties also outperform traditional varieties in terms of yield. Drought-tolerant varieties were tested in the northwestern part of Bangladesh and demonstrated better performance in adverse situations (Ahmed et al., 2017). As a result, rice farmers in those areas began to adopt these drought-tolerant rice varieties.

Adoption of drought-tolerant rice varieties can play an important role in agriculture sector development, maintaining food grain supply and improving the well-being of a substantial number of people. The decision to adopt, on the other hand, is complicated. Several factors may affect the decision, and identifying these factors is critical for the sector's future growth. According to Mottaleb et al. (2014), land characteristics, access to credit, infrastructure, and irrigation facilities have a significant effect on the adoption of modern rice varieties. Adoption of stress-tolerant rice varieties is influenced substantially by education (Ahmed et al., 2016). Cho and Kim (2019) find that household assets, credit, and involvement in farmers field school show a positive impact on the adoption of drought-tolerant rice varieties in the Philippines. A few studies (Kumar et al., 2008; Arouna and Aboudou, 2019) have assessed the production effect of drought-tolerant rice varieties worldwide and conclude that these varieties provide a higher yield than traditional varieties. Islam (2018) indicate that adoption of improved rice varieties increases household income and food grain availability.

It is evident from the preceding literature review that many studies identify the determinants of modern rice variety adoption and its impact on productivity. However, there is a dearth of study regarding the factors that influence the intensity of adoption of drought-tolerant rice varieties. Furthermore, to the best of our knowledge, no studies measure the welfare impacts of drought-tolerant rice varieties in South Asia, and specifically Bangladesh. Given the importance of rice farming in Bangladesh's economy, it is critical to comprehend the factors that influence the intensity of adoption, which can, in turn, increase production and ensure food security for the people of Bangladesh. It is important to identify the factors of adoption in order to make the best use of extension tools. The contribution of this study is twofold. First, this study makes a methodological contribution to the literature by using a two-limit Tobit model to identify the factors that influence adoption intensity. Second, using treatment effect models, this study assesses the welfare impacts in terms of food grain availability, income, and productivity. This study bridges knowledge gaps for policymakers, which will aid in the implementation of drought-tolerant rice farming policies in Bangladesh.

## 2 Material and methods

### 2.1 Data sources

**Figure 1:**
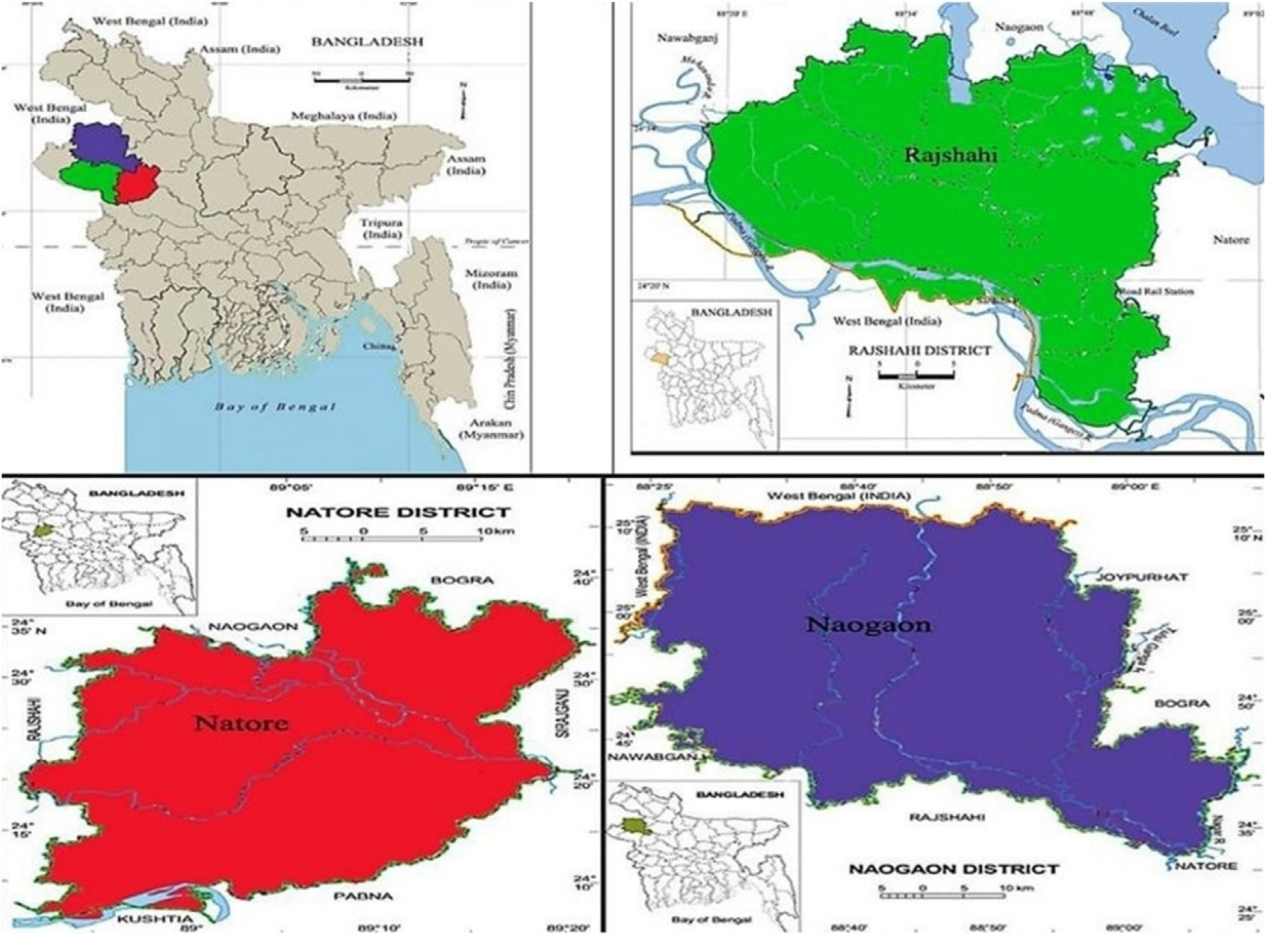
Map of study areas.

The sample for this study was selected using a multistage sampling technique. This study was conducted in three northwestern districts of Bangladesh: Rajshahi, Naogaon, and Natore (Figure 1). For the past three decades, Bangladesh's northwestern region has been suffering from drought (Habiba et al., 2013). Annual rainfall in these areas varies between 1,400 and 1,650 mm on average. According to several studies, the yearly total rainfall difference between the drought-affected region and the rest of Bangladesh is roughly 1,000 mm (Shahid and Hazarika, 2010; Habiba et al., 2013). During the summer, the average temperature in these areas frequently exceeds 40 °C. As a result of the high frequency of drought-related incidents, farmers in these three areas cultivate drought-tolerant rice. Thus, in the first stage, these three districts were selected for the study. In the second stage, in consultation with the local extension office, one sub-district was selected from each district. In the third stage, four villages were selected from each sub-district to conduct the face-to-face interviews. The following formula was used to determine the appropriate sample size (Kanyenji et al., 2020; Rahman et al., 2021a):

(1) $$n_{0}=\frac{z^{2}pq}{e^{2}}=\frac{{\left(1.96\right)}^{2}*0.5*0.5}{{\left(0.06\right)}^{2}}=267≅300$$

where *n*_*o*_ is the sample size, *z*^*2*^ is the 95% confidence interval, *p* is the estimated proportion of an attribute that is present in the population, *q* is 1−*p* and *e* is the desired precision level. First, a list of rice farmers was prepared for each village. From that list, 25 rice growers from each village were randomly selected for interview. Using Eq. (1), a total of 300 rice farmers were surveyed, with 100 from each district, using a pre-tested interview schedule.

The interview schedule was pre-tested with 20 rice farmers to verify that the questions contained in the schedule were clear and easy for respondents to answer. The responses of these 20 farmers were excluded from the final analysis. The interview schedule was finalized based on the rice farmers' recommendations and feedback. The final survey was conducted using an English-written paper-based interview schedule. To collect the data, three enumerators were employed and trained. The data was collected between February and March of 2021. Because the data collection took place during the COVID-19 outbreak, the enumerators used all of the essential protective gear and stayed at a safe distance. If any respondent stated that he or she was unwilling to participate in the interview, the data collectors selected an alternative from the farmers list as a sample. Out of 300 rice growers, 124 cultivated drought-tolerant rice varieties during Aman season (July–October 2020) and were thus classified as adopters. The remaining farmers were classified as non-adopters. Furthermore, to assess the welfare impacts, the 124 drought-tolerant rice varieties growers were divided into two groups: partial adopters (those who cultivated drought-tolerant varieties on a portion of their total rice cultivable land) and full adopters (those who cultivated drought-tolerant varieties on all of their total rice cultivable land). Out of 124 farmers, 89 were identified as partial adopters and 35 as full adopters of drought-tolerant rice varieties.

### 2.2 Analytical techniques

#### 2.2.1 Intensity of adoption

A Tobit regression model was used for identifying the factors influencing intensity of drought-tolerant rice varieties adoption. This model is commonly used where the range of the dependent variable is partially known (Wooldridge, 2010). Adoption of technology is rarely a simple yes or no decision (Noltze et al., 2012; Rahman et al., 2021b). As a result, a binary logit or probit model is insufficient for identifying the determinants that affect adoption intensity. To overcome this problem, several researchers use Tobit regression to estimate the adoption intensity (Martey et al., 2014; Paltasingh, 2018; Rodthong et al., 2020). Other researchers (Mal et al., 2012; Rahman et al., 2021b) employ the double hurdle model to investigate the factors that influence adoption and adoption intensity. The Tobit model was compared to the double hurdle model using a likelihood ratio (LR) test (Greene, 2003), and we selected the Tobit model to analyze the data based on the results of the LR test. In the Tobit regression model, the observed dependent variable $Y_{j}$ satisfies Eq. (2) and Eq. (3).

(2) $$Y_{j}=\max\left(Y_{j}^{*},0\right),$$

where, $Y_{j}^{*}$ represents a latent variable generated through the classical linear regression model.

(3) $$Y_{j}^{*}=\beta^{\prime }X_{j}+U_{j},Y_{j}=\left\{ \begin{array}{l} Y_{j}^{*}\;if\;Y_{j}^{*}>0\\ 0\;if\;Y_{j}^{*}\leq 0 \end{array}\right.$$

where $X_{j}$ represents independent variables, $\beta^{\prime }$ represents parameters to be estimated, and $U_{j}$ is expected to be normally distributed ($U_{j}∼N\left(0,\sigma^{2}\right)$ (Greene, 2003). The empirical latent variable model used to estimate the factors influencing intensity of drought-tolerant rice varieties adoption is specified as follows:

(4) $$Y_{j}^{*}=\beta_{0}+\beta_{1}X_{1j}+\beta_{2}X_{2j}+\beta_{3}X_{3j}+\ldots +\beta_{14}X_{14j}+\varepsilon_{j}.$$

The dependent variable of this model (Eq. (4)) is the intensity of drought-tolerant rice varieties (proportion of total area used for drought-tolerant rice varieties). Because censoring of the dependent variable occurred at both the upper and lower boundaries, a two-limit Tobit model was employed for this study (Rodthong et al., 2020). Before continuing with estimation of the model, the variance inflation factor (VIF) was calculated to identify multicollinearity. The calculated VIFs (mean VIF 1.63) were found to be less than 10, meaning that there was no multicollinearity (Maddala 1992). Based on extant literature and priori assumptions, a total of 14 independent variables were included in the model to predict their influences (Ali and Kumar, 2011; Noltze et al., 2012; Mariano et al., 2012; Ghimire et al., 2015; Chandio and Yuansheng, 2018; Rahman, 2022). Out of the 14 selected variables, education, family size, spouse education, severity of drought and distance from extension office were continuous variables, while the others were dummy variables. Descriptions of the independent variables used in the model are given in Table 1.

**Table 1:** Definition of the variables used in the models.

Variable		Notation	Description	Justification
Education (yrs)		*X*_1_	Total years of schooling of respondent.	Education provides the ability and capability to explore and work on new technology. It is expected that education will have a positive impact on the intensity of adoption.
Family size (No.)		*X*_*2*_	The total number of members in the family.	Farmers with larger family sizes prefer labor-intensive farming techniques such as rice farming. As a result, larger family sizes have a positive influence on the intensity of adoption.
Spouse education (yrs)		*X*_*3*_	Years of schooling of respondent's spouse.	An educated spouse assists their counterpart in making sound decisions, which may increase the likelihood of adoption.
Farm size (ha)		*X*_*4*_	The farm's total area in hectare.	Larger farms more likely to adopt than small ones.
Training (yes/no)		*X*_*5*_	1 if the respondent received training on farming related practices, otherwise 0.	Farmers become skilled and knowledgeable as a result of training. As a result, it has a positive impact on farmers' adoption decisions.
Access to credit (yes/no)		*X*_*6*_	1 if the respondent has access to formal credit, otherwise 0.	Adoption of technology necessitates expenses, and credit facilities can assist farmers by ensuring a steady flow of cash.
Membership (yes/no)		*X*_*7*_	1 if the respondent is a member in any society organization, otherwise 0.	Membership in a society organization expands the farmers' social network, which may positively increase adoption.
Health condition (yes/no)		*X*_*8*_	1 if the respondent is in good health, otherwise 0.	Farmers who are in good health are more likely to adopt new farming technologies.
Mobile phone (yes/no)		*X*_*9*_	1 if the respondent has a mobile phone, otherwise 0.	When a farmer has a mobile phone, he or she has the advantage of being able to communicate quickly and effectively with various agricultural service providers. Thus, this may positively influence adoption.
Television (yes/no)		*X*_*10*_	1 if the respondent watched agriculture-related TV shows, otherwise 0.	Television, for example, may be a useful source of information that might positively affect adoption decisions.
Severity of drought (Score)		*X*_*11*_	1 if the respondent faces low severity, 2 for moderate severity, 3 for high severity, and 0 for no severity. The data was then normalized as the perception of drought severity may vary among respondents. The normalized value of severity was used in the model.	Level of adoption may rise as the severity of drought rises.
Distance from extension office (km)		*X*_*12*_	Distance of respondent's house from local agricultural extension office.	Agricultural extension workers offer advice and solutions to farmers on a variety of agricultural challenges. Farmers that reside close to an extension office may readily communicate with extension workers and thus, be more likely to adopt.
Location dummy 1		*X*_*13*_	1 if the primary farmer is from Rajshahi, 0 otherwise.	The level of adoption may differ across locations. Two location dummies were used to avoid the dummy trap. As a reference category, Naogaon was used.
Location dummy 2		*X*_*14*_	1 if the primary farmer is from Natore, 0 otherwise.	

#### 2.2.2 Impact assessment

Impact analysis of this study involved multiple groups of responses like non-adopters, partial adopters and full adopters. To identify the non-linearities and differential effects among the treatments, multivalued treatment effect (MVTE) models, regression adjustment (RA), inverse probability weighting (IPW), and inverse probability weighted regression adjustment (IPWRA) were used (Cattaneo, 2010; Wooldridge, 2010). Wooldridge (2010) estimates the cases through explaining the participation in a training program which may occur at different levels like part-time or full-time. Cattaneo (2010) develops a theory for semiparametric estimators and applies that to analyze quantile treatment effects. The MVTE models have conditional independence and sufficient overlap assumption (Ma et al., 2021). In addition, MVTE models work through observed characteristics; as a result, bias for unobservable characteristics may exist (Kazal et al., 2020). These requirements were fulfilled through introducing a large set of independent variables (Table 1) into the treatment model.

RA, a simple extension of binary to multivalued cases, estimates the potential effect of treatment without any prior assumption on the treatment model (Wooldridge, 2010). A two-step approach was used in the RA model. First, a separate outcome model was estimated for each treatment level. Second, the average treatment effect on the treated (ATT) was estimated by using differences of potential outcomes.

Extension of the binary case to the multivalued cases also suggests the use of inverse probability weighting (IPW) estimator. This helps avoid the extrapolation and selection bias problem and thus, improves the covariate balance (Mansournia and Altman, 2016). IPW was estimated through two steps. In the first step, the propensity score was estimated using a multinomial logistic regression and in the second step, inverse of the propensity score was used as weights in calculating the average value of the outcome variable (Imbens, 2004).

Inverse probability weighted regression adjustment (IPWRA), alternatively known as Wooldridge's ‘doubly robust’ estimator (Wooldridge, 2010), ensures consistent and unbiased results since it allows the treatment and the outcome model to account for misspecification (Ma et al., 2021). In the IPWRA model, ATT was estimated through two steps (Imbens and Wooldridge, 2009). First, propensity scores were estimated through multinomial logistic regression; then, ATT was computed through a linear regression model.

The impact was assessed using three outcome variables: productivity, household income, and food grain availability from own production. Productivity is the amount of rice produced per hectare of land in one year. The total income received from agricultural and off-farm sources in one year is referred to as annual income. Annual income was calculated in USD (1 USD = Tk. 85, Tk. being the Bangladeshi currency). Farmers were asked how many months they could consume from their own production in a year in order to assess food grain availability.

## 3 Results and discussion

### 3.1 Descriptive statistics

It is evident from Table 2 that the socioeconomic and farm characteristics are almost identical for all three groups of farmers. Out of 300 farmers, about 59% were classified as non-adopters, while only 11.67% were full adopters, indicating that farmers were reluctant to adopt drought-tolerant rice varieties. Merely 11% of non-adopters underwent agricultural-related training, while about 50% of adopters did. The findings also indicate that full adopters, on average, live closer to an extension office (3.81 km) than partial and non-adopters. Farmers that reside close to an extension office may be able to communicate more effectively with extension officers, allowing them to learn about new technology and adopt it more quickly. Agricultural fairs are frequently held on the premises of extension offices, which may also facilitate farmers in increasing their awareness of new technology (Rahman, 2021). The findings also reveal that more training programs should be implemented in the study areas, particularly for farmers who are not adopting drought-tolerant rice varieties. This may assist them in gaining a thorough understanding of the technology, resulting in a higher rate of adoption (Adegbola and Gardebroek, 2007). The average farm size for the three groups of farmers is nearly identical. The majority of full and partial adopters possess modern communication devices such as mobile phones, which can help them contact neighboring farmers and extension staff, and thereby increase adoption rates of new technology (Rahman et al., 2021c). Full adopters have faced more extreme drought than partial and non-adopters, which may have influenced their decision to adopt.

**Table 2:** Descriptive statistics of the variables.

Variables	Full adopters		Partial adopters		Non-adopters	
	Mean	Standard deviation	Mean	Standard deviation	Mean	Standard deviation
Education (yrs)	4.89	4.12	5.61	4.68	5.17	4.87
Family size (No.)	4.46	1.22	5.63	5.87	5.78	3.04
Spouse education (yrs)	5.51	4.42	6.71	6.95	4.88	4.35
Farm size (ha)	0.93	0.63	0.96	0.88	0.98	1.20
Training (yes/no)	0.49	0.51	0.55	0.50	0.11	0.32
Access to credit (yes/no)	0.43	0.50	0.38	0.49	0.36	0.48
Membership (yes/no)	0.49	0.51	0.46	0.50	0.50	0.50
Health condition (yes/no)	0.43	0.50	0.42	0.50	0.44	0.50
Mobile phone (yes/no)	0.66	0.48	0.58	0.50	0.44	0.51
Television (yes/no)	0.86	0.36	0.89	0.32	0.69	0.46
Severity of drought (Score)	0.33	0.26	0.19	0.22	0.16	0.24
Distance from extension office (km)	3.81	3.49	5.98	4.21	5.45	3.78
Location dummy 1	0.63	0.49	0.22	0.42	0.33	0.47
Location dummy 2	0.34	0.48	0.62	0.49	0.19	0.39
Observations	35		89		176	

### 3.2 Factors affecting intensity of adoption

The findings of the two-limit Tobit model are presented in Table 3. The significant LR chi-square value indicates that the model is appropriate for the sampled data. The results of the Tobit model indicate that training, farmer's health condition, ownership of mobile phone, ownership of television and location all have a positive influence on the intensity of adoption, while society membership has a negative influence on the adoption (Table 3).

**Table 3:** Factors affecting intensity of adoption.

Variable	Coefficients	SE	p-value
Education (yrs)	-0.011	0.013	0.398
Family size (No.)	-0.002	0.013	0.853
Spouse education (yrs)	0.007	0.010	0.479
Farm size (ha)	0.041	0.060	0.499
Training (yes/no)	0.791∗∗∗	0.130	0.000
Access to credit (yes/no)	-0.079	0.119	0.504
Membership (yes/no)	-0.311∗∗	0.126	0.014
Health condition (yes/no)	0.274∗∗	0.126	0.030
Mobile phone (yes/no)	0.195∗	0.116	0.092
Television (yes/no)	0.363∗∗	0.158	0.023
Severity of drought (Score)	1.412	1.059	0.183
Distance from extension office (km)	-0.015	0.014	0.281
Location dummy 1	0.522	0.562	0.353
Location dummy 2	1.080∗∗∗	0.173	0.000
Constant	-1.488∗∗∗	0.283	0.000
Log pseudolikelihood	-205		
LR chi-square	132∗∗∗		
Pseudo R^2^	0.24		
Number of observations	300		

Note: ∗, ∗∗, and ∗∗∗ indicates significant at 10%, 5%, and 1% level, respectively.

The positive and significant coefficient of training indicates that intensity of adoption is higher for trained farmers than untrained farmers. This finding is consistent with the findings of Zakaria et al. (2020) and Aryal et al. (2018). The majority of farmer training in Bangladesh is provided through government extension offices. Crop cultivation, insect and disease management, and postharvest operations are all covered in the trainings. These trainings are expected to greatly improve rice farmers' awareness and, as a result, raise level of adoption. Training brings farmers into contact with professionals who have a wide range of expertise, which may boost their knowledge and skills and hence the intensity of adoption. The positive effect of training on adoption of a new technology is well documented (Rahman et al., 2018; Rahman, 2021). The importance of training in technology adoption has been reinforced by the findings in this study.

The negative relationship between society membership and adoption implies that the intensity of adoption is lower among farmers who are members of any society organization compared to their counterparts. Several studies (Wossen et al., 2017; Massresha et al., 2021; Rahman, 2021) find that society membership affects agricultural technology adoption favorably. It is envisaged that social organizations would overcome information gaps and minimize the cost of exploring new technologies. However, in our research, we find the inverse association. We are unable to pinpoint the reasons for this result since we lacked information on the nature of the society organizations. It is assumed that not all of the society organizations considered in our study are agricultural in nature. As a result, farmers active in non-agricultural organizations may not receive adequate information about new agricultural technology, which may discourage adoption. Previous research also shows that involvement in society organizations can decrease the likelihood of agricultural technology adoption (Neupane et al., 2002; Tigabie et al., 2013; Kazal et al., 2020).

Farmers' health condition affects their adoption intensity of drought-tolerant rice varieties. Farmers in good health can work more intensely on the farm, which may boost the adoption intensity of new farming technology. Farmers' health has also been demonstrated to have a positive impact on agricultural technology adoption in previous studies (Li et al., 2012b, Li et al., 2021a). If a farmer becomes ill, money may be diverted away from farming to cover healthcare costs, thus hindering the implementation of new technologies (Lipton et al., 2002). It is also expected that if farmers are in good health, they will be able to communicate with extension workers and other farmers, as well as participate in awareness-raising activities such as demonstrations and field days organized by various government and non-government organizations, all of which will increase the intensity of adoption.

The coefficient of mobile phone ownership indicates that farmers who own their own mobile phone adopt drought-resistant rice varieties more than their counterparts. Information plays a critical role in a farmer's decision to adopt a technology (Mittal and Mehar, 2016; Chandio and Yuansheng, 2018). Farmers with mobile phones can develop their skills and expertise by communicating with other farmers and extension staff. Farmers need up-to-date knowledge on recently produced and released varieties, and mobile phone connectivity provides them with this ability.

The findings of this study indicate that technology-based information resources, such as television, have a substantial influence on intensity of adoption. Intensity of adoption is higher in farmers who watch agriculture-related TV shows. A positive and significant coefficient value of television implies that households with access to television will have greater access to knowledge and awareness about climate change and climate-smart agricultural practices. There are national and regional programs that broadcast information on climate-smart agricultural activities in this regard. As a result, individuals who have access to information through various communication channels are more likely to adopt new technology than their counterparts (Shallo et al., 2020). This may mean that it is past time to switch up the extension methods. Mass media may cover a larger geographical area at a lower cost and may also play an important role in cultivating farmer understanding and knowledge, which could promote increased adoption.

Furthermore, the probability of adopting drought-tolerant rice was higher in the Natore district compared to the Rajshahi and Naogaon districts, which may be attributed to the fact that farmers in these two districts experienced less drought compared to farmers in the Natore district.

### 3.3 Welfare impacts

Table 4 shows that both partial and full adoption of drought-tolerant rice varieties has a substantial influence on farmers' food grain availability. Based on various treatment effect models, ATT values show that full adopters can consume 1.02–1.29 months more from their own production in a year than non-adopters (Table 4). The impact of full adoption ranges from 9.80% to 12.64 %. The results of all three alternative models are nearly identical, showing the robustness of the finding. Farmers who partially adopt drought-tolerant rice varieties can consume 0.71–0.81 months more from their own production in a year than non-adopters, based on the IPWRA and RA models, respectively. The data also show that there is a substantial difference in food grain availability between partial and full adopters, implying that farmers who grow drought-tolerant rice varieties on all of their rice cultivable land are in a better position in terms of food grain availability. Adoption of drought-tolerant rice varieties helps to increase food grain supply, which can play an important role in food security (Mottaleb et al., 2017; Kader et al., 2019; Dar et al., 2020).

**Table 4:** Impact on food grain availability.

Category of farmers	RA			IPW			IPWRA			
	ATT	Robust SE	% higher than PO mean	ATT	Robust SE	% higher than PO mean	ATT	Robust SE	% higher than PO mean	
Partial adopters vs Non-adopters	0.85∗	0.46	8.31	0.63	0.41	6.11	0.71∗	0.42	6.96	
Full adopters vs Non-adopters	1.21∗	0.71	11.88	1.02∗∗	0.41	9.80	1.29∗∗	0.55	12.64	
Full adopters vs Partial adopters	0.60	0.59	5.51	0.81∗	0.42	7.58	1.07∗∗	0.42	10.00	

Note: ∗, and ∗∗ indicates significance at the 10%, and 5% level, respectively; PO indicates potential outcome.

Table 5 shows that both partial and full adoption of drought-tolerant rice varieties have a substantial influence on productivity. Farmers who fully or partially adopt drought-tolerant rice varieties outperform non-adopters in terms of yield. This conclusion is consistent with the findings of Mottaleb et al. (2017) and Dar et al. (2020). According to the ATT values, rice farmers who grow drought-tolerant cultivars on all of their rice cultivable area yield 1222–1473 kg more per hectare than non-adopters. Dar et al. (2020) estimates that farmers who adopt drought-tolerant rice varieties in drought-stressed Indian regions enjoy more than a ton of yield per hectare more than non-adopters. According to the ATT values, rice farmers who partially adopt drought-tolerant varieties obtain 725, 761, and 786 kg more yield per hectare than non-adopters based on the RA, IPW, and IPWRA models, respectively. Based on several treatment effect models, the ATT value also suggests that full adopters obtain 716–1089 kg more yield per hectare than partial adopters. Dar et al. (2020) finds that drought-tolerant varieties provide better yields during non-drought years. Li et al., 2012b, Li et al., 2021a also finds that drought-tolerant rice varieties yield as much as conventional high-yielding rice varieties under normal conditions in China. Selvaraj et al. (2010) also discovers that farmers who adopt drought-tolerant rice varieties profit by more than 20% due to the combination of cost efficiency and increased production. According to the findings of previous research, drought-tolerant rice varieties outperform traditional varieties even in non-drought years. Therefore, full adoption of drought-tolerant rice varieties is crucial for enhancing productivity and increasing food grain availability in drought-prone areas.

**Table 5:** Impact on rice productivity.

Category of farmers	RA			IPW			IPWRA		
	ATT	Robust SE	% higher than PO mean	ATT	Robust SE	% higher than PO mean	ATT	Robust SE	% higher than PO mean
Partial adopters vs Non-adopters	725∗∗∗	195	17.13	761∗∗∗	190	18.14	786∗∗∗	180	18.85
Full adopters vs Non-adopters	1267∗∗∗	297	29.94	1473∗∗∗	234	44.65	1222∗∗∗	270	29.31
Full adopters vs Partial adopters	716∗∗∗	272	13.78	1089∗∗∗	308	21.59	727∗∗∗	184	14.09

Note: ∗∗∗ indicates significance at the 1% level.

According to this study's findings, full adopters have a higher income than non-adopters, which is consistent with the findings of Selvaraj et al. (2010). Based on various treatment effect models, the impact on income ranges from 3.46% to 4.22% (Table 6). Yamano et al. (2018) find that farmers in India benefit from the adoption of a short-duration drought-tolerant rice variety by producing the following crop early. Farmers may be able to sell more on the market as a result of increased productivity, resulting in a greater income. Thus, farmers may be able to diversify their income sources. Although partial adopters show better productivity than non-adopters, their income is not significantly different. This might be because full adopters have nearly double the production of partial adopters, allowing them to make more money by selling rice in the market and diversifying their income sources. As a result, full adopters have considerably higher incomes than non-adopters. Farmers' economic losses may also be reduced as a result of adoption (Kader et al., 2019). Concerned authorities should take appropriate strategies to communicate and improve farmers' knowledge, which will boost the adoption intensity of drought-tolerant rice varieties.

**Table 6:** Impact on yearly income.

Category of farmers	RA			IPW			IPWRA		
	ATT	Robust SE	% higher than PO mean	ATT	Robust SE	% higher than PO mean	ATT	Robust SE	% higher than PO mean
Partial adopters vs Non-adopters	-0.07	0.08	-0.58	-0.06	0.08	-0.50	-0.10	0.09	-0.83
Full adopters vs Non-adopters	0.45∗∗∗	0.13	3.75	0.52∗∗∗	0.09	4.22	0.43∗∗∗	0.12	3.46
Full adopters vs Partial adopters	0.52∗∗∗	0.08	4.26	0.64∗∗∗	0.09	5.24	0.51∗∗∗	0.07	4.18

Note: ∗∗∗ indicates significance at the 1% level.

## 4 Conclusions

Drought is likely to be a key issue for sustainable rice production as a result of the long-term negative effects of climate change. This study uses cross-section data to identify the factors of the intensity of adoption of drought-tolerant rice varieties and its impact on farmer welfare in Bangladesh. Approximately 41% of the farmers surveyed adopted drought-tolerant rice varieties, with about 11.67% being classified as full adopters. According to the findings of this research, training and technology-related variables such as ownership of a mobile phone and exposure to agriculture-related television shows all play a major role in the decision to adoption drought-resistant rice varieties. A number of policy implications can be taken from this study's results. Intensity of adoption can be greatly increased through the use of mass media such as television. Modifying the extension approach through mass media and other communication strategies will aid in widespread adoption of new technologies because these technology-based approaches can increase awareness. Programs about climate change and other relevant topics should be broadcast through the mass media on a regular basis to raise awareness. Since farmer health is a major factor in adoption, concerned authorities can develop a policy intervention mechanism to provide minimal health care facilities at the farm level, which will, in turn, provide better health care facilities for the farmers, increasing their work efficiency and willingness to adopt new technologies. This study also emphasizes the importance of the government's role in farmer training and raising consciousness about adoption. Long-term programs, such as training on climate-smart technologies, are needed to boost adoption. Adoption of drought-tolerant rice varieties enhances food grain availability, increases income, and improves productivity. As a result, farmers will be less sensitive to climate-related shocks such as drought. Higher productivity raises the prospect of higher income, which may then be utilized to generate money from other sources, thus alleviating poverty in drought-prone areas.

## Declarations

### Author contribution statement

Md. Sadique Rahman: Conceived and designed the experiments; Performed the experiments; Analyzed and interpreted the data; Contributed reagents, materials, analysis tools or data.

Md. Hayder Khan Sujan: Analyzed and interpreted the data; Contributed reagents, materials, analysis tools or data; Wrote the paper.

Debasish Chandra Acharjee: Analyzed and interpreted the data; Wrote the paper.

Rezoyana Kabir Rasha; Mofasser Rahman: Contributed reagents, materials, analysis tools or data; Wrote the paper.

### Funding statement

This work was supported by the Sher-e-Bangla Agricultural University Research System(SAURES).

### Data availability statement

Data will be made available on request.

### Declaration of interests statement

The authors declare no conflict of interest.

### Additional information

No additional information is available for this paper.
